# Contagious Impetigo

**Published:** 1897-04

**Authors:** Wm. S. Gottheil


### CONTAGIOUS IMPETIGO.

This is a self-limited contagious disease of children, appearing in localized epidemics, and first described by Tilbury Fox, n 1864. Accompanied by a moderate fever and some gastric disturbance, there appear on the face and hands groups of flat vesicles filled with transparent or cloudy serum. These dry up into characteristic golden-yellow crusts, which fall off in two or three weeks, leaving circular, reddened, non-ulcerated areas behind. Successive crops of vesicles may prolong the disease for two months or more. It is undoubtedly parasitic, but, though Kaposi claims to have found it, the etiological factor is still unknown. The treatment consists in removal of the crusts with olive-oil compresses, cleansing the skin with hot water and soap, boric acid solution, etc., followed by the use of Lassar's paste:

℞—Acid salicylic............................30 grains.

Petrolati................................ 1 ounce.

Zinoi oxidi.... ....................... } aa. ½ ounce.

Amyli.. ... ........................ }

WM. S. GOTTHEIL, M. D., in *Pediatrics*, October, 1896.

